# Prognostic and clinical implications of CT-morphometric sarcopenia in adult myxofibrosarcoma patients: a longitudinal analysis

**DOI:** 10.1186/s12957-025-04069-6

**Published:** 2025-10-28

**Authors:** Julian Kylies, Alonja Reiter, Elias Brauneck, Jana K. Striefler, Karl-Heinz Frosch, Matthias Priemel

**Affiliations:** 1 Department of Trauma and Orthopedic Surgery, University Medical Center Hamburg Eppendorf, Martinistraße 52, Hamburg, 20246 Germany; 2 Department of Oncology, Hematology and Bone Marrow Transplantation With Section Pneumology, Hubertus Wald University Cancer Center, University Medical Center Hamburg-Eppendorf, Hamburg, Germany; 3 Department of Trauma Surgery, Orthopaedics and Sports Traumatology, BG Klinikum Hamburg, Hamburg, Germany

## Abstract

**Background:**

Myxofibrosarcoma (MFS) is a rare, aggressive soft tissue sarcoma with a high local recurrence rate, mostly affecting elderly patients. Sarcopenia, the progressive loss of skeletal muscle mass, and its CT-related assessment is an emerging prognostic factor in cancer care, yet its role in MFS remains unclear.

**Methods:**

We conducted a retrospective longitudinal study of 55 patients with high-grade MFS who underwent surgical resection and had at least two CT scans between 2010 and 2024 with a mean scan-interval of 13.2 ± 2.1 months. CT-derived morphometric parameters, including skeletal muscle index (SMI), paraspinal muscle index (PSMI), psoas muscle index (PMI), skeletal muscle density (SMD), and visceral adipose tissue (VAT), were measured at the L3 vertebral level. We analyzed changes in body composition over time and their associations with chemotherapy, radiotherapy, tumor recurrence, postoperative complications, survival and functional status.

**Results:**

SMI, PSMI, PMI, and VAT declined significantly during the disease course, while SMD remained stable. Chemotherapy and local tumor recurrence were associated with greater muscle and fat loss, whereas radiotherapy showed no significant impact. A ≥ 15% decrease in SMI was associated with shorter median overall survival (32 vs. 80 months, *p* = 0.02). Although pre-treatment sarcopenia did not affect survival it was linked to longer hospital stays (*p* = 0.03) and increased risk for postoperative wound infections (*p* = 0.01).

**Conclusion:**

CT-based body composition analysis offers a practical approach for risk stratification in MFS patients. Routine assessment of sarcopenia using existing imaging can help identify those at higher risk for poor survival, extended hospitalization, and wound complications. Incorporating these metrics into preoperative planning may guide early supportive measures, such as nutritional and wound care interventions. Prospective multicenter studies are needed to confirm these findings and assess whether targeted strategies can reduce sarcopenia-related morbidity and improve outcomes in this rare sarcoma.

## Introduction

Myxofibrosarcoma (MFS) is a malignant soft tissue tumor of fibroblastic origin, characterized by its myxoid stroma and infiltrative growth pattern [1, 2]. Although predominantly affecting elderly patients, it can occur across all age groups and poses significant therapeutic challenges due to its high risk for local recurrence despite wide surgical margins and adjuvant therapies such as chemo-/radiotherapy [3–5].

Sarcopenia [6], defined by the progressive loss of skeletal muscle mass and function, has emerged as a crucial prognostic factor in oncologic care. Its presence is associated with increased chemotherapy toxicity, prolonged hospital stays, postoperative complications (e.g. wound infections), and reduced survival across various malignancies, including colorectal and lung cancer [7–11]. Although sarcopenia is commonly associated with aging and chronic illnesses, it can also result from disease-induced metabolic stress and the cumulative burden of intensive oncologic treatments, such as chemotherapy and major surgical interventions [12–14]. Traditional assessment methods, including clinical tests like handgrip strength and gait speed, offer valuable functional insights but are often time-consuming and may be impractical for routine clinical use [15]. Furthermore, these tools may not adequately reflect the extent of body composition alterations driven by both aging and tumor-related processes.

Advances in cross-sectional imaging have enabled the assessment of body composition using standard CT scans routinely used in both preoperative planning and postoperative care [16]. Parameters such as the skeletal muscle index (SMI), psoas muscle index (PMI), paraspinal muscle index (PSMI), skeletal muscle density (SMD), and visceral adipose tissue (VAT) can be reliably measured at the L3 vertebral level, providing detailed insight into patient sarcopenia and metabolic reserve [16–18].

While the prognostic relevance of CT-derived body composition metrics has already been established for many solid tumors, including colorectal, and lung cancer, little is known about their prognostic value or clinical implications in soft tissue sarcomas [19–21]. Particularly MFS, a relatively rare and biologically heterogeneous malignancy remains understudied in this regard. Given its tendency to affect elderly patients and its locally aggressive nature, body composition changes during treatment may hold important prognostic value. To date, no study has longitudinally evaluated how morphometric CT parameters evolve over the disease course in MFS or how these changes relate to treatment response, complications, functional decline, or survival.

To address this gap, we conducted a retrospective longitudinal analysis of CT-derived morphometric parameters in patients with high-grade MFS undergoing surgical resection. Specifically, we aimed to characterize how skeletal muscle mass and quality as well as visceral adipose tissue change over the course of the disease and treatment. We investigated how systemic and local therapies, such as chemotherapy and radiotherapy, influence these body composition parameters. Additionally, we examined whether changes in CT-related body composition parameters are associated with overall survival, functional status and postoperative complications. By utilizing routinely acquired CT scans, this study offers new insight into the clinical and prognostic relevance of CT-related sarcopenia in MFS patients and highlights its potential role as a prognostic and treatment-related marker in this rare but aggressive malignancy.

## Materials and methods

This retrospective study was approved by the local ethics committee (ID: 2025–300576-WF) and conducted in accordance with the Declaration of Helsinki. Due to the anonymized and retrospective design of the study, the requirement for informed consent was waived.

**Fig. 1:**
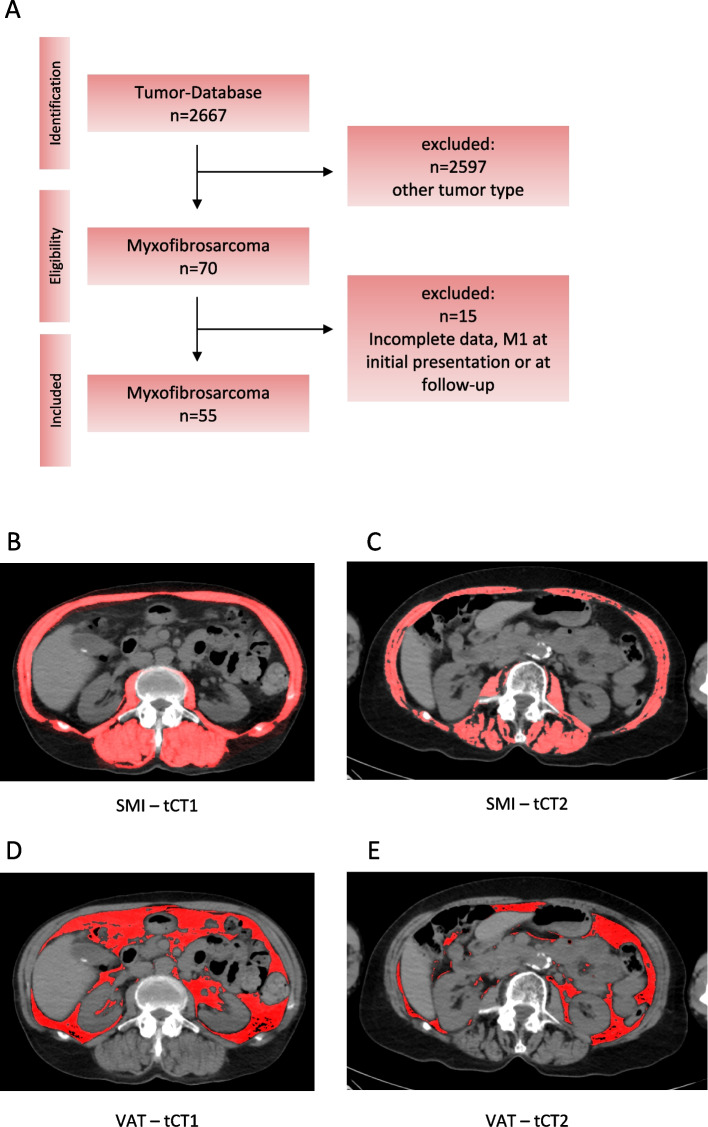
Study design and CT morphometric assessment of muscle and visceral adipose tissue over the disease course. Figure 1A shows the patient selection process as well as the study design (**A**). Skeletal muscle index (SMI) and visceral adipose tissue (VAT) were measured at baseline (tCT1) and follow-up (tCT2), revealing a progressive decline in both SMI (**B**–**C**) and VAT (**D**–**E**) during the observation period

A total of 55 patients with a histologically confirmed diagnosis of high-grade MFS who underwent surgical resection between 2010 and 2024 were included. All patients had at least two consecutive CT scans: the first performed prior to surgical resection and the second obtained at a minimum follow-up interval of 12 months. These scans served as defined time points (tCT1 and tCT2) for longitudinal assessment of CT-based morphometric changes. The patient selection process is illustrated in Fig. 1A. CT scans were acquired exclusively on the same scanner (Siemens SOMATOM Definition AS, Siemens Healthineers, Erlangen, Germany) at the University Medical Center Hamburg-Eppendorf, ensuring methodological consistency.

Patients were eligible for inclusion if they had a baseline TNM classification of N0M0, high-quality CT scans with clear visualization of the L3 vertebral level, and complete clinical data, including ECOG (Eastern Cooperative Oncology Group) performance status, resection margin status, TNM classification, chemotherapy regimen, radiotherapy, available at both imaging time points. Additional data included surgical site infection rate and length of hospital stay following surgical resection.

Patients were excluded if they had fewer than two CT scans, insufficient image quality due to motion artifacts, metal implants, or incomplete coverage of the L3 level, or if they had prior or concurrent malignancies that could interfere with morphometric analysis. Additionally, patients with a history of spinal surgery or musculoskeletal disorders such as muscular dystrophy or neuromuscular conditions were excluded. Incomplete clinical or laboratory data preventing robust longitudinal analysis also led to exclusion.

CT morphometric parameters were assessed using Fiji imaging software (Version 2.3.0/1.53q, Max Planck Institute of Molecular Cell Biology and Genetics, Dresden, Germany) with a semi-automated thresholding approach. For each patient, a single axial slice at the mid-L3 vertebral level was selected. Using the polygon selection tool, regions of interest (ROIs) were manually outlined for the total skeletal musculature, paraspinal muscles, and psoas muscles. Following ROI definition, tissue segmentation was performed by applying standardized Hounsfield unit (HU) thresholds ranging from –29 to + 150 HU to exclude non-muscular tissue. The cross-sectional areas (cm^2^) of skeletal muscle, paraspinal muscles, and psoas muscles were then normalized to patient height squared (cm^2^/m^2^) to derive the SMI, PSMI, and PMI [17, 22]. SMD, representing muscle quality, was determined by calculating the mean HU within the segmented paraspinal muscle ROI. VAT was measured on the same slice by applying a threshold range of –190 to –30 HU and manually delineating the intra-abdominal compartment, excluding subcutaneous fat (Fig. 1).

Clinical and laboratory parameters were extracted retrospectively from electronic medical records at both time points tCT1 and tCT2.

### Statistical analysis

Data analysis was conducted using IBM SPSS Statistics v.29 (IBM, Armonk, NY, USA). Graphical visualizations were generated using GraphPad Prism v.10.2.2 (GraphPad Software, *La Jolla, CA, USA*). Changes in CT morphometric body composition parameters across time points were evaluated using the non-parametric Mann–Whitney-U test. Continuous data are presented as mean ± standard deviation (SD). Statistical significance was set at *p* < 0.05.

### Survival analysis

Survival outcomes were assessed using Kaplan–Meier curves to compare survival probabilities between sarcopenic and non-sarcopenic patients at baseline (defined by SMI) [23, 24], as well as between patients with ≥ 15% vs. < 15% reductions in SMI over the disease course. For survival analyses, only patients between 2010 and 2020 were included into the analysis to ensure a follow-up of at least 5 years. Differences between survival curves were tested using the log-rank (Mantel-Cox) test. To determine the optimal cut-off value for SMI changes predictive of survival, a Receiver Operating Characteristic (ROC) curve analysis was performed. SMI measurements were obtained at the first and last time points, and the percentage change in SMI between these time points was calculated for each patient. The optimal cut-off value was identified using Youden’s Index (J). This analysis determined that a 15% decrease in SMI represented the most predictive threshold, with patients experiencing such a reduction exhibiting significantly worse survival outcomes than those with smaller declines.

### Cox proportional hazards regression analysis

To evaluate whether changes in SMI from tCT1 to tCT2 serves as an independent predictor of survival, a Cox proportional hazards regression model was constructed. The model was adjusted for age, sex, ECOG performance status, local tumor recurrence, tumor diameter and chemotherapy exposure. For overall survival, we fitted Cox proportional hazards models with pre-treatment covariates (age, sex, ECOG, tumor size, depth, resection margin status, tumor site, radiotherapy) and chemotherapy. Where chemotherapy initiation occurred after tCT1, it was modelled as a time-dependent covariate. For longitudinal body-composition outcomes, we used ANCOVA models of percentage change between tCT1 and tCT2, adjusting for baseline values of each CT metric and the same pre-treatment covariates, including chemotherapy. Local recurrence, a post-baseline event, was not adjusted for in models estimating chemotherapy effects to avoid over-adjustment. Sensitivity analyses using propensity-score weighting for chemotherapy were performed to assess robustness. All patients included in the survival analysis had a TNM status of N0 and M0 at baseline. Cox regression was performed using partial likelihood estimation, with the Efron method applied for handling ties. Hazard ratios (HRs) with 95% confidence intervals (CIs) were reported for all covariates. Statistical significance was set at *p* < 0.05.

## Results

### Basic demographic data and study design

Using our institutional tumor registry, a total of 2,667 patients diagnosed with sarcomas between 2010 and 2024 were screened. Of these, 2,597 patients with non-MFS malignancies were excluded, resulting in an initial cohort of 70 patients with a histologically confirmed diagnosis of grade 3 MFS. After further exclusion of 15 patients due to missing CT imaging, incomplete clinical or laboratory data, or metastatic disease at initial presentation, 55 patients (21 female) met the final inclusion criteria. The mean age of the study cohort was 71 ± 13.1 years. Baseline demographic characteristics are summarized in Table 1.

**Table 1:** Patient characteristics at baseline

	Male	Female
Total (n)	34	21
Mean age (years)	71 ± 13.1	73 ± 13.3
Mean follow up (months)	12.9 ± 3.2	13.4 ± 2.0
Local tumor recurrence (LR)	16 (47%)	11 (52%)
Surgery only	11 (32%)	7 (33%)
Surgery and radiation	12 (35%)	8 (38%)
Surgery and chemotherapy	8 (24%)	5 (24%)

Illustrated are the baseline characteristics of the patient cohort

The mean interval between CT scan 1 (tCT1) and CT scan 2 (tCT2) was 13.2 ± 2.1 months, allowing sufficient time to detect meaningful changes in CT-based morphometric parameters over the disease course. The first CT scan was performed prior to surgery, with a mean interval of 0.4 ± 0.2 months between imaging and surgical resection. The second CT scan was obtained postoperatively after a mean of 12.6 ± 2.2 months, in accordance with standardized institutional and national follow-up and surveillance guidelines Table 2.

**Table 2:** CT-morphometric and clinical parameters over the disease course

	Mean tCT1 (SD)	Mean tCT2 (SD)
Skeletal Muscle Index (SMI) [cm^2^/m^2^]	44.3 (5.0)	33.6 (7.0)
Male	47.6 (4.8)	34.3 (7.6)
Female	40.9 (5.1)	32.7 (6.3)
Paraspinal Muscle Index (PSMI) [cm^2^/m^2^]	16.6 (0.9)	10.1 (2.7)
Male	17.9 (1.0)	10.6 (2.8)
Female	16.1 (0.8)	9.6 (2.5)
Psoas Muscle Index (PMI) [cm^2^/m^2^]	2.5 (0.5)	1.1 (0.6)
Male	2.6 (0.6)	1.1 (0.5)
Female	2.5 (0.5)	1.2 (0.6)
Skeletal Muscle Density (SMD) [25]	42.5 (4.0)	41.3 (7.1)
Male	42.9 (4.1)	40.8 (8.5)
Female	42.0 (3.9)	42.6 (7.7)
Visceral Adipose Tissue (VAT) [cm^2^]	80.3 (5.7)	62.3 (2.9)
Male	84.5 (6.1)	65.5 (3.0)
Female	76.0 (5.3)	59.1 (2.7)
ECOG	1.0 (2.0)	1.0 (3.0)

Illustrated are CT-morphometric and clinical parameters and the changes over the disease course

*Abbreviations*: *ECOG* Eastern Cooperative Oncology Group

### Longitudinal decline in CT morphometric body composition parameters in male and female MFS patients

Across the entire cohort of MFS patients, a significant decline was observed in CT morphometric body composition parameters between tCT1 and tCT2, indicating progressive deterioration of muscle mass and visceral adiposity over the disease course.

**Fig. 2:**
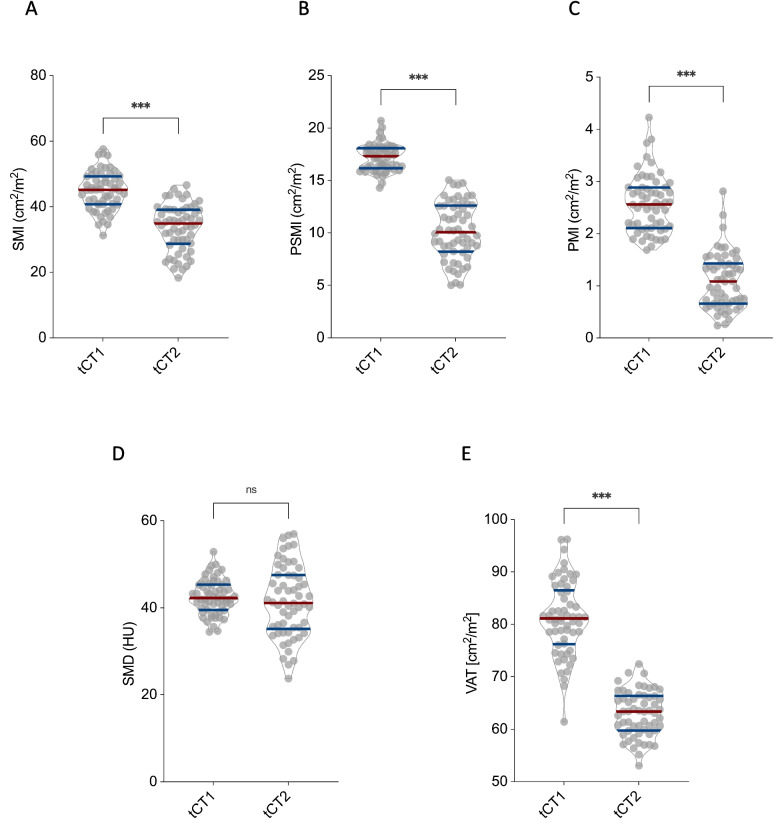
Longitudinal changes in CT-morphometric parameters in myxofibrosarcoma patients. Depicted are the longitudinal changes in skeletal muscle mass, skeletal muscle density (SMD), and visceral adipose tissue (VAT) across the entire cohort of myxofibrosarcoma patients between tCT1 and tCT2. Skeletal muscle index (SMI) (**A**), paraspinal muscle index (PSMI) (**B**), psoas muscle index (PMI) (**C**), and VAT (**E**) all showed significant declines over the disease course (*p* < 0.001), whereas SMD (**D**) remained stable without significant changes (*p* = 0.33)

SMI significantly decreased from 44.9 ± 5.9 to 33.7 ± 7.1 cm^2^/m^2^ (*p* < 0.001; Fig. 2A). PSMI declined from 16.7 ± 1.4 to 10.1 ± 2.7 cm^2^/m^2^ (*p* < 0.001; Fig. 2B), and PMI from 2.6 ± 0.5 to 1.1 ± 0.5 cm^2^/m^2^ (*p* < 0.001; Fig. 2C). In contrast, SMD remained stable, with values of 42.5 ± 4.0 at tCT1 and 41.5 ± 8.1 at tCT2 (*p* = 0.33; Fig. 2D). VAT showed a significant reduction, decreasing from 81.1 ± 7.1 to 63.0 ± 4.3 cm^2^ (*p* < 0.001; Fig. 2E).

Taken together, these results highlight a robust and consistent loss of skeletal muscle mass and visceral fat in MFS patients over the disease course, while muscle quality as measured by SMD appeared unchanged.

### Impact of chemotherapy and radiotherapy on longitudinal changes in CT-morphometric body composition

To investigate the impact of adjuvant therapies on body composition, changes in CT morphometric parameters between tCT1 and tCT2 were analyzed across three patient groups: those undergoing surgery only, surgery with chemotherapy, and surgery with local radiotherapy. 4 patients who underwent surgery, chemotherapy and radiation were excluded. Chemotherapy was considered for patients with high-risk localized MFS (tumor size ≥ 5 cm, deep (subfascial) location, high risk of R1 resection or close margins, neurovascular involvement, rapid growth, or early local recurrence after prior surgery).

**Fig. 3:**
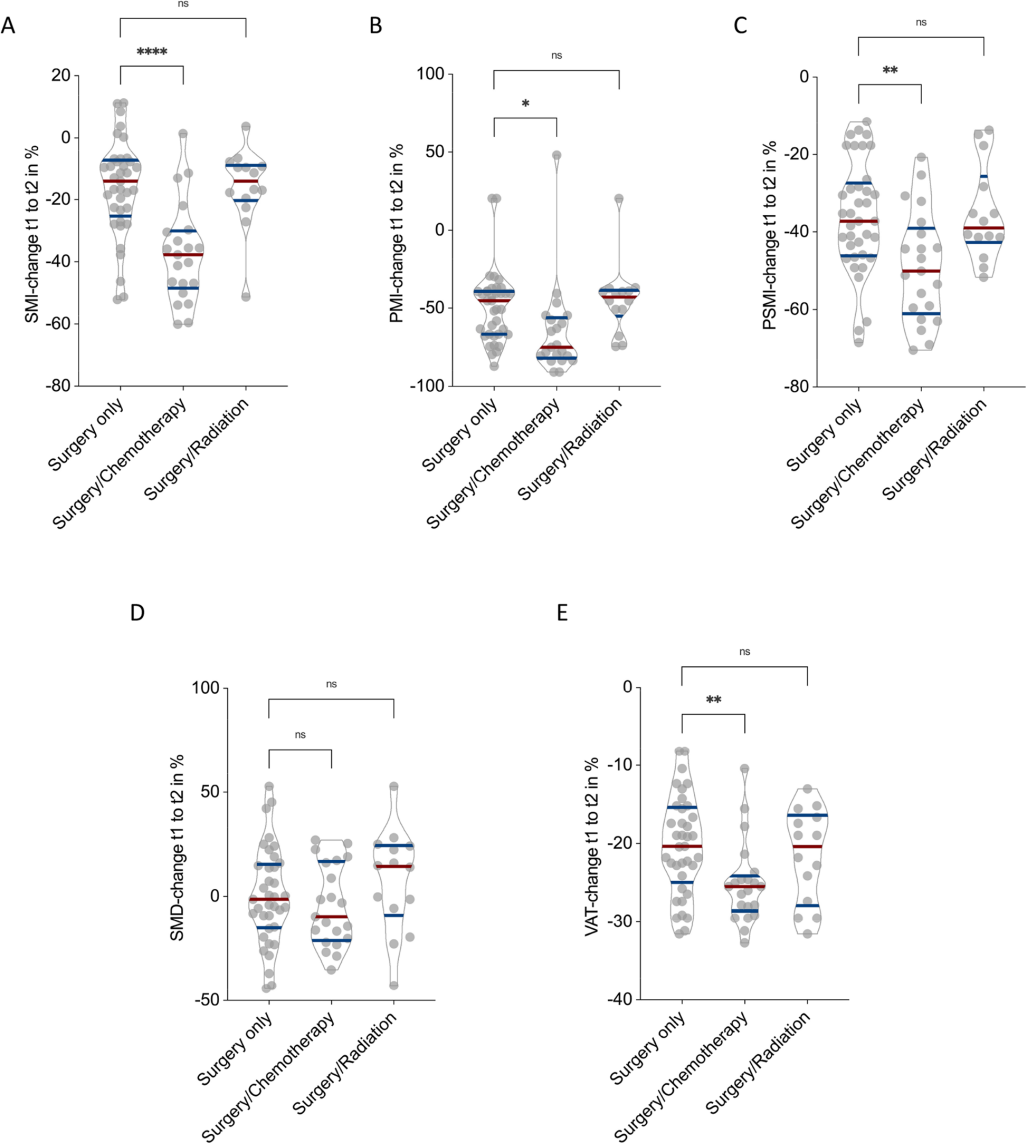
Impact of chemotherapy and radiotherapy on changes in CT morphometric parameters Between tCT1 and tCT2 in Myxofibrosarcoma Patients. Illustrated is the percentage changes in Skeletal muscle index (SMI) (**A**), Paraspinal muscle index (PSMI) (**B**), Psoas muscle index (PMI) (**C**), Skeletal muscle density (SMD) (**D**), and Visceral adipose tissue (VAT) (**E**) stratified by treatment group: surgery only, surgery with chemotherapy, and surgery with radiotherapy. Patients receiving chemotherapy exhibited significantly greater declines in SMI (*p* < 0.0001), PSMI (*p* = 0.02), PMI (*p* < 0.01), and VAT (*p* < 0.01) compared to those undergoing surgery alone. No significant differences were observed for SMD (*p* = n.s.) or for any parameter in the surgery and radiotherapy group

Patients who received both surgery and chemotherapy exhibited significantly greater declines in skeletal muscle and adipose tissue parameters compared to those treated with surgery alone. Specifically, SMI declined by an average of −37.2 ± 7.1% in the chemotherapy group versus −16.1 ± 6.9% in the surgery-only group (*p* < 0.0001, Fig. 3A). Similarly, PSMI and PMI decreased more markedly in patients receiving chemotherapy (−48.9 ± 6.1% and −65.2 ± 5.9%, respectively) than in those with surgery only (−36.0 ± 5.1% and −49.1 ± 4.9%, respectively), with statistically significant differences (*p* = 0.02 for PSMI, *p* < 0.01 for PMI; Fig. 3B and C). VAT also showed a more pronounced reduction in the chemotherapy group (−25.1%) compared to the surgery-only group (−20.2 ± 5.5%), reaching statistical significance (*p* < 0.01, Fig. 3E). No significant differences in SMD were observed between these two groups (*p* = n.s., Fig. 3D). In contrast, the addition of local radiotherapy to surgery did not significantly affect CT morphometric decline. Patients in this group showed changes in SMI, PSMI, PMI, SMD, and VAT that were comparable to those observed in patients who underwent surgery alone (all *p* = n.s.).

Collectively, these findings show that chemotherapy is associated with a significantly greater deterioration in muscle mass and visceral fat in MFS patients, while local radiotherapy does not appear to exert a comparable effect on CT-based body composition parameters.

### Influence of local tumor recurrence on CT morphometric body composition parameters

To assess the impact of local tumor recurrence (LR) on changes in CT morphometric body composition, patients were stratified based on the presence or absence of tumor recurrence between tCT1 and tCT2.

**Fig. 4:**
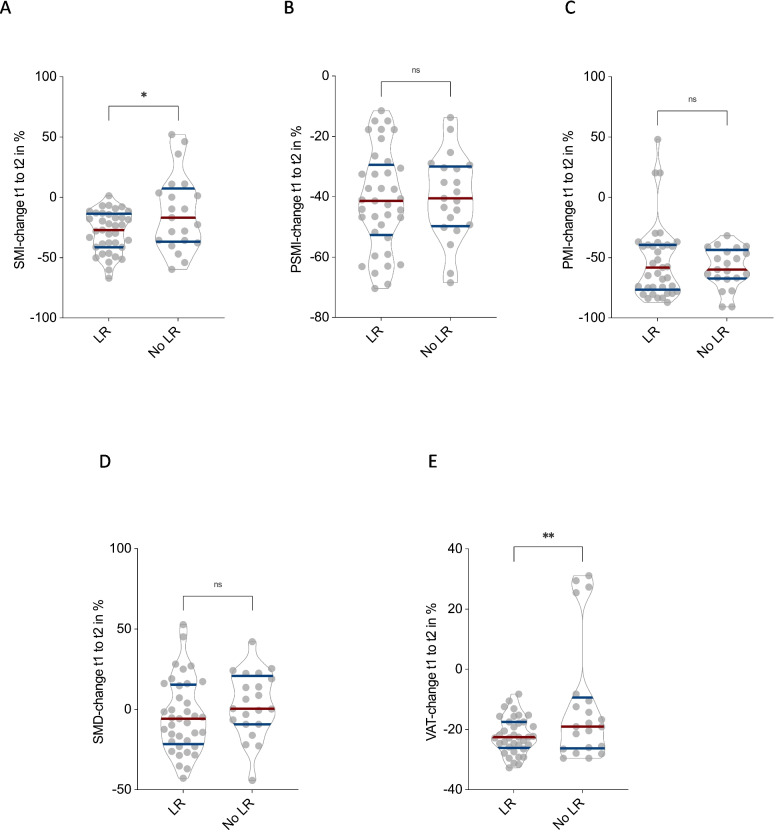
Association of local tumor recurrence with CT morphometric body composition changes in myxofibrosarcoma patients. Comparison of percentage changes in Skeletal muscle index (SMI) (**A**), Paraspinal muscle index (PSMI) (**B**), Psoas muscle index (PMI) (**C**), Skeletal muscle density (SMD) (**D**), and Visceral adipose tissue (VAT) (**E**) between patients with and without local tumor recurrence (LR). Significantly greater reductions in SMI (*p* = 0.02) and VAT (*p* < 0.01) were observed in patients with local tumor recurrence, while changes in PSMI, PMI, and SMD did not differ significantly

Patients with LR showed significantly greater declines in SMI (–28.5 ± 4.4%) compared to those without recurrence (no LR) (–12.5 ± 3.7%, *p* = 0.02; Fig. 4A). Similarly, VAT was significantly more reduced in the LR group (–21.9 ± 3.9%) than in patients with no LR (–11.4 ± 3.8%, *p* < 0.01; Fig. 4E). For PSMI, PMI, and SMD, no statistically significant differences were observed between the two groups (all *p* = n.s.; Fig. 4B–D).

These findings suggest that local tumor recurrence may contribute to more pronounced loss of overall skeletal muscle mass and visceral adipose tissue, while regional muscle indices and muscle quality appear to be less affected.

### Influence of tumor localization on CT morphometric body composition parameters

To evaluate whether tumor localization influences longitudinal changes in CT morphometric parameters, patients were stratified into two groups: those with tumors located in the lower extremities or trunk (*n* = 36) and those with tumors of the upper extremities (*n* = 19).

**Fig. 5:**
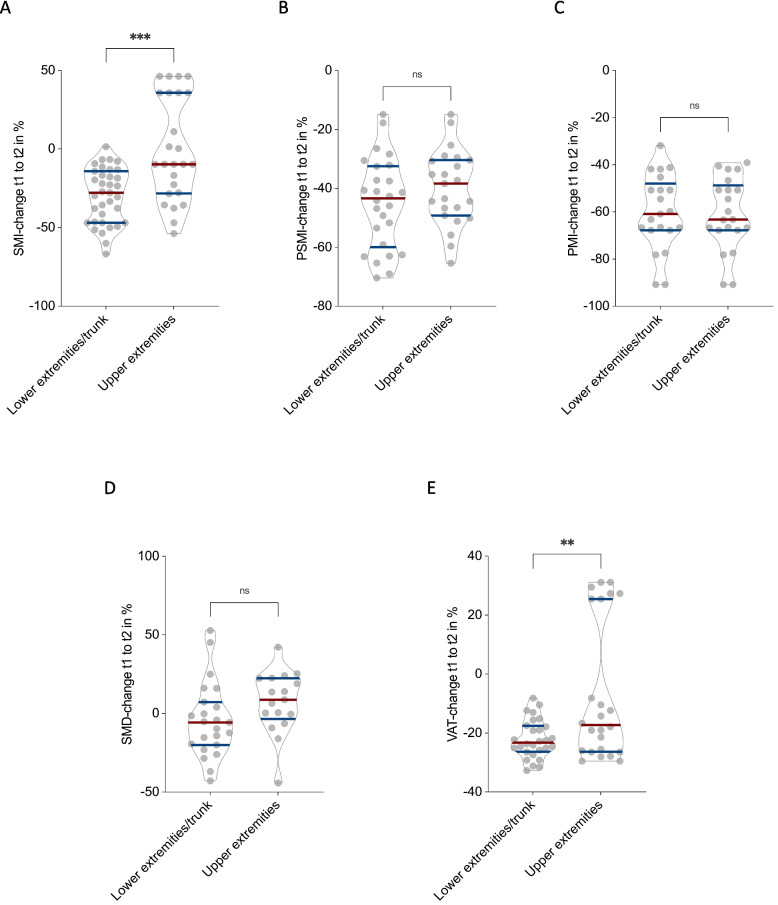
Influence of tumor localization on CT-morphometric body composition. Longitudinal changes in body composition between tCT1 and tCT2 stratified by tumor site: lower extremities/trunk (*n* = 36) vs. upper extremities (*n* = 19). Patients with lower extremity/trunk tumors showed significantly greater declines in SMI (**A**, *p* < 0.001) and VAT (**E**, *p* < 0.01), while no significant differences were observed for PSMI (**B**), PMI (**C**), or SMD (**D**)

Patients with tumors in the lower extremities or trunk demonstrated significantly greater declines in skeletal muscle and adipose tissue compared to patients with tumors of the upper extremities. Specifically, SMI decreased by –29.7 ± 17.5% in the lower extremity/trunk group, whereas patients with upper extremity tumors showed almost no decline (–0.5 ± 32.8%, *p* < 0.001; Fig. 5A). Similarly, VAT decreased significantly more in the lower extremity/trunk group (–22.1 ± 6.5%) compared to the upper extremity group (–6.7 ± 23.7%, *p* < 0.01; Fig. 5E). In contrast, no statistically significant differences were observed for PSMI (–44.9 ± 15.4% vs. –39.7 ± 12.8%, *p* = 0.22; Fig. 5B), PMI (–60.2 ± 16.0% vs. –60.7 ± 15.4%, *p* = 0.41; Fig. 5C), or SMD (–4.3 ± 23.7% vs. 7.3 ± 19.9%, *p* = 0.31; Fig. 5D).

These findings indicate that tumor localization in the lower extremities or trunk is associated with more pronounced losses in overall skeletal muscle mass and visceral adipose tissue, potentially reflecting the greater impact of impaired mobilization in these patients.

### Prognostic impact of CT morphometric muscle loss on overall survival

To evaluate the prognostic significance of longitudinal changes in skeletal muscle mass, ROC curve analysis with Youden’s J statistic was performed to determine an optimal threshold for SMI decline predictive of survival. A 15% reduction in SMI between tCT1 and tCT2 was identified as the most discriminative cut-off.

**Fig. 6:**
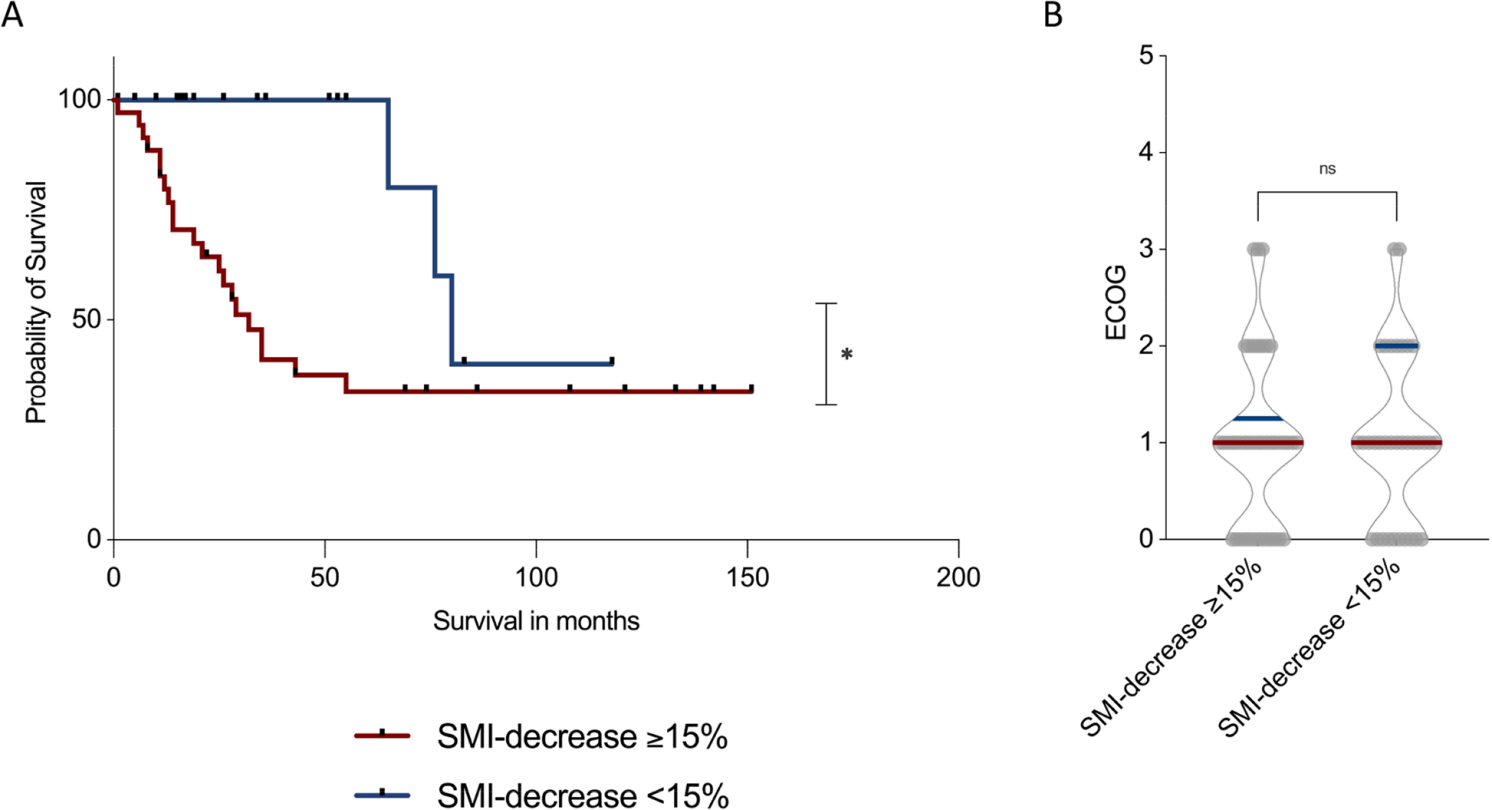
Prognostic impact of decline in skeletal muscle index (SMI) on survival and functional status in myxofibrosarcoma patients. Kaplan–Meier survival analysis comparing patients with ≥ 15% vs. < 15% reduction in SMI (**A**). A significant difference in overall survival was observed between groups (*p* = 0.02). ECOG performance status at follow-up did not significantly differ between groups (**B**)

Patients who experienced a ≥ 15% decrease in SMI had significantly shorter median overall survival (32 months) compared to those with < 15% SMI loss (80 months; *p* = 0.02; Fig. 6A).

However, no significant differences in functional status, as measured by ECOG performance score, were observed between the groups. Both cohorts had a median ECOG score of 1, with similar ranges (ECOG < 15%: range 3; ECOG ≥ 15%: range 3; Fig. 6B).

To further assess the prognostic relevance of individual clinical and morphometric factors, univariate and multivariate Cox proportional hazards regression models were performed.

In univariate Cox analyses, a ≥ 15% decrease in SMI was associated with reduced OS (HR 1.45; 95% CI 1.10–2.15; *p* = 0.015). Additional factors associated with poorer OS included age (HR 1.03; 95% CI 1.01–1.08; *p* < 0.01), local tumor recurrence (HR 2.47; 95% CI 1.09–5.11; *p* = 0.017), tumor diameter (HR 1.21; 95% CI 1.05–1.27; *p* < 0.01), ECOG (HR 1.37; 95% CI 1.21–1.79; *p* < 0.001), and chemotherapy exposure (HR 1.31; 95% CI 1.22–2.15; *p* = 0.02); sex was not significant (Table 3).

**Table 3:** Univariate Cox hazard regression model

Predictor	Hazard Ratio	95%-Confidence Interval	*p*-Value
Age	1.029	1.01–1.08	< 0.01**
Sex	0.79	0.33–1.81	0.541
Local tumor recurrence	2.47	1.09–5.11	0.017*
Tumor diameter	1.21	1.05–1.27	< 0.01**
ECOG	1.37	1.21–1.788	< 0.001***
SMI-decrease	1.45	1.10–2.15	0.015*
Chemotherapy	1.31	1.22–2.15	0.02*

Illustrated are the results of the univariate cox hazard regression model

*Abbreviations*: *ECOG* Eastern Cooperative Oncology Group, *SMI* Skeletal Muscle Index

*p <0.05, **p <0.01, ***p <0.001

In the multivariate Cox model, a ≥ 15% SMI decrease remained an independent predictor of worse OS (HR 1.41; 95% CI 1.07–2.11; *p* = 0.011), together with local recurrence (HR 2.43; 95% CI 1.04–4.12; *p* = 0.028), tumor diameter (HR 1.21; 95% CI 1.02–1.25; *p* < 0.01), ECOG (HR 1.20; 95% CI 1.11–1.81; *p* < 0.01), and chemotherapy (HR 1.25; 95% CI 1.19–2.45; *p* = 0.02). Age and sex were not independently associated with OS after adjustment (Table 4).

**Table 4:** Multivariate Cox hazard regression model

Predictor	Hazard Ratio	95%-Confidence Interval	*p*-Value
Age	1.022	0.881–1.331	0.441
Sex	0.841	0.407–1.767	0.667
Local tumor recurrence	2.425	1.041–4.121	0.028*
Tumor diameter	1.211	1.022–1.251	< 0.01**
ECOG	1.201	1.109–1.811	< 0.01**
SMI-decrease	1.411	1.069–2.112	0.011*
Chemotherapy	1.25	1.19–2.45	0.02*

Illustrated are the results of the multivariate cox hazard regression model

*Abbreviations*: *ECOG* Eastern Cooperative Oncology Group, *SMI* Skeletal Muscle Index

*p <0.05, **p <0.01

Together, these results emphasize the independent prognostic value of longitudinal SMI loss alongside classical clinical factors.

### Impact of preoperative sarcopenia on survival, functional status, and postoperative complications

To investigate the clinical relevance of preoperative sarcopenia in MFS patients, survival, functional outcomes, and postoperative complications were compared between sarcopenic and non-sarcopenic patients. Sarcopenia was defined using established sex-specific SMI cut-off values (< 52.4 cm^2^/m^2^ for males and < 38.5 cm^2^/m^2^ for females) [23, 24].

**Fig. 7:**
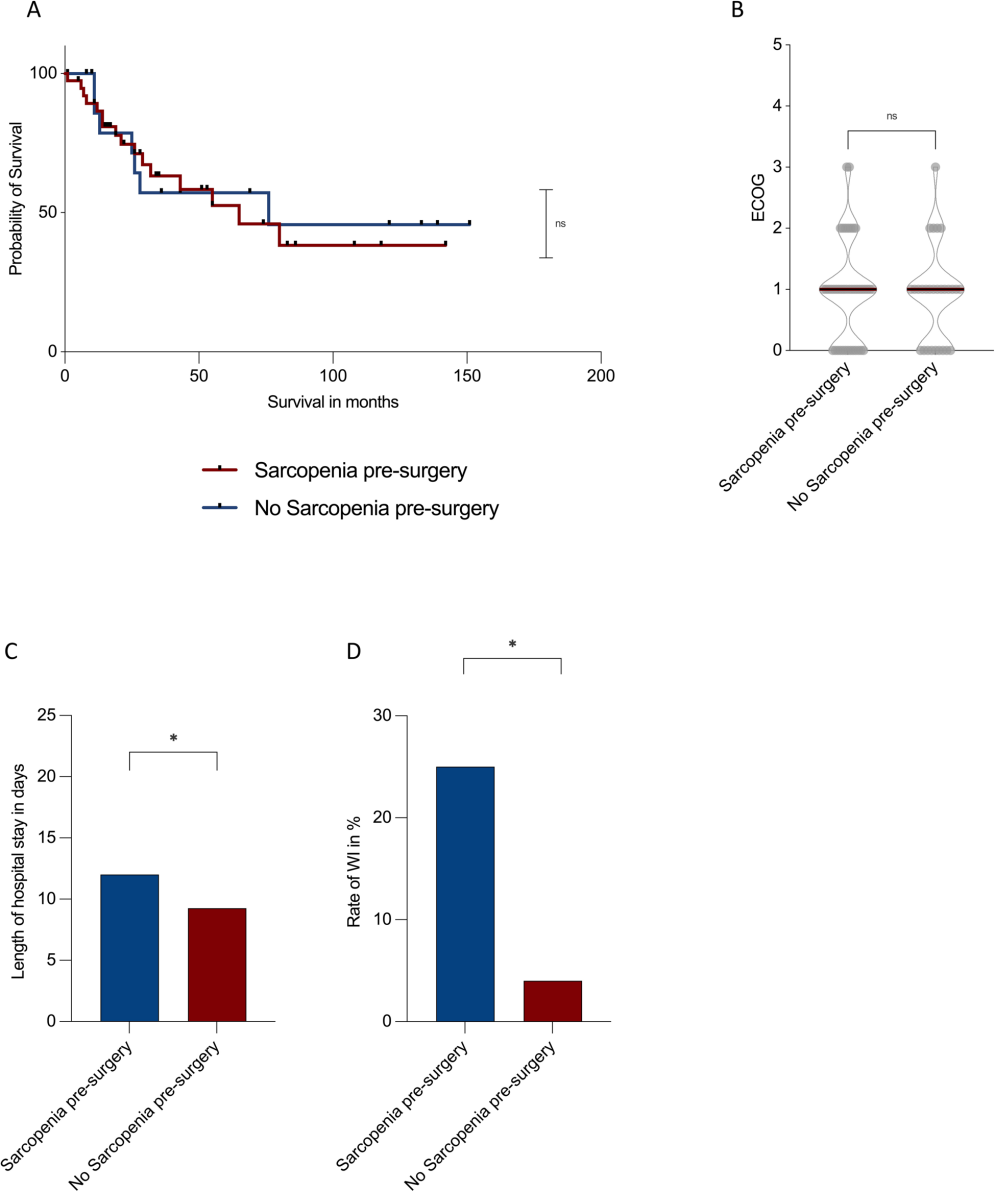
Effect of preoperative sarcopenia on survival, functional status, and postoperative outcomes in myxofibrosarcoma patients. Comparisons between sarcopenic and non-sarcopenic patients regarding overall survival (**A**), ECOG performance status (**B**), length of hospital stay (**C**), and rate of postoperative wound infections (**D**). No significant differences were found for survival (*p* = 0.44) or ECOG status (*p* = 0.71). Sarcopenic patients had significantly longer hospital stays (*p* = 0.03) and higher wound infection rates (*p* = 0.01)

No significant difference in OS was observed between sarcopenic and non-sarcopenic patients. The median survival in the sarcopenic group was 65 months, compared to 76 months in the non-sarcopenic group (*p* = 0.44; Fig. 7A). Similarly, preoperative ECOG performance status did not differ significantly between groups, with a median ECOG score of 1 and comparable ranges (range: 3) in both sarcopenic and non-sarcopenic patients (*p* = 0.71; Fig. 7B).

However, sarcopenic patients experienced a significantly longer postoperative hospital stay (12.0 ± 4.1 days vs. 9.3 ± 2.2 days, *p* = 0.03; Fig. 7C). Furthermore, the rate of postoperative wound infections requiring antibiotic therapy or revision surgery was significantly higher in sarcopenic patients (25%) compared to non-sarcopenic patients (4%; *p* = 0.01, Fig. 7D). Patients without primary surgical closure (e.g., vacuum-assisted wound therapy) were excluded from this analysis to avoid confounding due to prolonged hospitalization.

These findings suggest that while preoperative sarcopenia may not significantly influence long-term survival or functional status in MFS patients, it is associated with a more complicated postoperative course, including longer hospital stays and higher rates of wound complications.

## Discussion

This study provides novel insights into the dynamics of CT-derived morphometric body composition parameters in patients with high-grade MFS. Our data show a significant decline in skeletal muscle indices (SMI, PSMI, PMI) and VAT over the disease course, while SMD remained stable. These changes were more pronounced in patients undergoing chemotherapy or experiencing local tumor recurrence. Furthermore, a ≥ 15% reduction in SMI between two consecutive CT scans was significantly associated with reduced overall survival. Preoperative CT-related sarcopenia, although not predictive of survival, was associated with longer hospital stays and higher postoperative wound infection rates. Together, these findings suggest that both baseline and longitudinal CT morphometric parameters may offer clinically meaningful insights into patient risk stratification and outcomes in MFS.

The consistent decline in skeletal muscle mass and visceral fat over the disease course in our cohort suggests a systemic, treatment- and disease-related deterioration in body composition. Sarcopenia in cancer patients is often driven by multiple factors including inflammatory cytokines, endocrine dysfunction, and tumor-induced metabolic alterations [26, 27]. In MFS patients, who often undergo extensive surgical resection procedures and receive adjuvant systemic therapies, these factors likely contribute to accelerated muscle wasting and fat depletion. The observed stability in SMD over time may indicate that while muscle mass is lost, muscle quality is preserved, at least over the time frame of observation. Our findings are in line with results from other malignancies, such as gastrointestinal and thoracic cancers, where quantitative loss in muscle and fat are prominent [28, 29] over the disease course, and support the broader relevance of sarcopenia across diverse tumor entities.

Next, our results reveal a clear impact of chemotherapy on body composition, with significantly greater reductions in SMI, PSMI, PMI, and VAT in patients undergoing perioperative chemotherapy compared to those treated with surgery alone or surgery and local radiotherapy. These findings are consistent with the known catabolic effects of cytotoxic agents such as anthracycline-based chemotherapy commonly used in MFS-treatment [30, 31] and the metabolic stress imposed by such treatment regimens. In contrast, patients treated with local radiotherapy did not exhibit significantly greater declines in CT-related body composition parameters, suggesting that localized treatments may not contribute substantially to systemic sarcopenia. Additionally, patients with local tumor recurrence experienced significantly greater reductions in both SMI and VAT, pointing to the potential role of tumor-associated inflammation and functional impairment in exacerbating muscle and fat loss. These patients typically require additional surgical resections and are more likely to receive intensified systemic therapy, both of which may further contribute to the development of sarcopenia. These data underline the importance of monitoring body composition in patients receiving intensive therapy or exhibiting early signs of recurrence. In addition, our analysis revealed that tumor localization influences longitudinal changes in body composition. Patients with tumors in the lower extremities or trunk experienced significantly greater declines in SMI and VAT compared to those with tumors of the upper extremities, suggesting that impaired mobilization may exacerbate frailty in this subgroup as well.

Furthermore, our findings demonstrate that a ≥ 15% reduction in SMI over the disease course is significantly associated with shorter overall survival in MFS patients. This highlights the prognostic value of longitudinal changes in skeletal muscle mass, suggesting that dynamic body composition metrics may serve as more sensitive indicators of clinical deterioration than static baseline measurements. In line with this, baseline sarcopenia, defined using established sex-specific SMI cut-off values [23, 24], was not associated with long-term survival or functional status but was significantly linked to prolonged postoperative hospital stay and a higher rate of wound infections. These findings align with those observed in other malignancies such as colorectal and lung cancer, where preoperative sarcopenia has been independently associated with increased risk of surgical complications, longer inpatient recovery, and impaired wound healing [32–34]. Furthermore, both CT-derived and clinically assessed sarcopenia have repeatedly been shown to predict poor OS in various solid tumors, underlining its relevance as an oncologic biomarker [7–11]. Together, our results emphasize the dual clinical significance of sarcopenia in MFS, both as a dynamic predictor of prognosis and as a baseline risk factor for perioperative complications.

To the best of our knowledge, this study provides the first longitudinal analysis of CT-derived body composition changes in patients with high-grade MFS, offering novel insights into the clinical relevance of sarcopenia in this rare soft tissue malignancy. A major strength of our study is the use of routinely acquired CT imaging, enabling a standardized and reproducible assessment of skeletal muscle and adipose tissue metrics without requiring additional imaging or patient burden. Furthermore, the integration of clinical outcomes including survival, ECOG performance, wound complications, and hospital stay allowed for a comprehensive evaluation of both oncologic and perioperative relevance.

However, several limitations should be acknowledged. First, the retrospective design and relatively small sample size, inherent to the rarity of MFS, may limit the generalizability of the findings and introduce potential selection bias. While CT-based measurements were performed using standardized thresholds at the L3 vertebral level, variability in imaging protocols and scanner calibration across the study period cannot be fully excluded. Furthermore, while baseline TNM status and treatment modalities were adjusted for residual confounding from unmeasured variables -including nutritional status, systemic inflammation profiles, or comorbid conditions- may have contributed to the observed body composition dynamics. Another limitation is that wound closure techniques (e.g., flap coverage or skin grafts) were not incorporated into multivariate models due to the low number of patients undergoing such procedures. Since these reconstructive approaches are common in superficial MFS and may independently affect wound complication rates, our results on the association between sarcopenia and wound healing should be interpreted with caution. Finally, while we identified meaningful associations between sarcopenia and survival or complications, the study does not establish causality. Because chemotherapy is preferentially offered to patients with higher baseline risk, we adjusted for chemotherapy in multivariable models and, where applicable, as a time-dependent exposure in survival analyses. These adjustments mitigate—but cannot entirely eliminate—confounding by indication. Consistency of results across conventional adjustment and propensity-weighted sensitivity analyses supports the robustness of the associations. Prospective validation in larger, multicenter cohorts will be necessary to confirm these findings and define their clinical utility.

CT-derived body composition analysis holds promise as a practical tool for risk stratification in MFS patients. Routine evaluation of sarcopenia using existing imaging may help identify individuals at increased risk for adverse outcomes such as poor survival, prolonged hospitalization, and wound complications. Integrating these metrics into preoperative planning could inform early supportive interventions such as nutritional support and wound care planning. Future prospective multicenter studies are needed to validate these findings and determine whether targeted strategies can mitigate sarcopenia-related morbidity and improve outcomes in this rare sarcoma entity.

## Data Availability

All data will be made available upon request.
